# Inducing Dose Sparing with Inactivated Polio Virus Formulated in Adjuvant CAF01

**DOI:** 10.1371/journal.pone.0100879

**Published:** 2014-06-23

**Authors:** Jes Dietrich, Lars Vibe Andreasen, Peter Andersen, Else Marie Agger

**Affiliations:** 1 Department of Infectious Disease Immunology, Statens Serum Institut, Copenhagen, Denmark; 2 Department of Vaccine Development, Statens Serum Institut, Copenhagen, Denmark; Federal University of São Paulo, Brazil

## Abstract

The development of new low cost inactivated polio virus based vaccines (IPV) is a high priority, and will be required to eradicate polio. In addition, such a vaccine constitutes the only realistic polio vaccine in the post-eradication era. One way to reduce the cost of a vaccine is to increase immunogenicity by use of adjuvants. The CAF01 adjuvant has previously been shown to be a safe and potent adjuvant with several antigens, and here we show that in mice IPV formulated with CAF01 induced increased systemic protective immunity measured by binding and neutralization antibody titers in serum. CAF01 also influenced the kinetics of both the cellular and humoral response against IPV to produce a faster, as well as a stronger, response, dominated by IgG2a, IgG2b, and IgG2c isotypes as well as IPV specific T cells secreting IFN-γ/IL-2. Finally, as intestinal immunity is also a priority of polio vaccines, we present a vaccine strategy based on simultaneous priming at an intradermal and an intramuscular site that generate intestinal immune responses against polio virus. Taken together, the IPV-CAF01 formulation constitutes a new promising vaccine against polio with the ability to generate strong humoral and cellular immunity against the polio virus.

## Introduction

Poliomyelitis is caused by the polio virus, an RNA virus that can colonize the gastroenteral tract which may lead to an acute, viral, infectious disease that spreads from person to person, primarily via the fecal-oral route. In 1988, the World Health Assembly resolved to globally eradicate poliomyelitis (polio) [1]. The initial objective, the end of polio by 2000, has proven more difficult than originally envisioned and polio still exist in countries such as Afghanistan, Nigeria and Pakistan. However, due to great efforts the number of polio cases has decreased to a level where full eradication within a decade or two is a realistic goal.

Two vaccines exist against polio; Inactivated polio Vaccine (IPV) and Trivalent live Oral polio Virus (tOPV). tOPV with attenuated Sabin strains of poliovirus types 1, 2 and 3, has been the vaccine of choice for polio vaccination in most countries because it induces both systemic and intestinal immunity, can immunize or boost immunity of close contacts through secondary spread, and is inexpensive and easy to administer. However, one problem with OPV is that on rare occasions OPV can cause vaccine-associated paralytic poliomyelitis (VAPP) and/or can revert to a neurovirulent form of poliovirus which is believed to be as transmissible and virulent as wild polioviruses [1]–[3]. Therefore, steps have been taken to discontinue OPV as a vaccine against polio, rendering IPV the only realistic polio vaccine in the post-eradication era.

When OPV is withdrawn, several challenges concerning IPV have to be dealt with. One such challenge is that the high purchase costs for IPV potentially can lead to limited supplies of IPV in many countries. Another challenge concerns the immunity induced by IPV, and how to achieve intestinal immunity with this vaccine. IPV protects the vaccine recipient from paralysis, but compared to OPV it provides less protection against re-infection. Furthermore IPV does not reduce fecal excretion following re-infection as much as OPV because it provides weaker intestinal immunity [4]–[8]. There are however studies that demonstrated that IPV can induce some intestinal immunity [5]–[7].

One way to reduce the cost of a vaccine is to use adjuvants [9]. In the field of pandemic influenza vaccines the use of adjuvants has permitted dose reduction, increased the availability and reduced cost of the vaccine [10]–[14]. Therefore, it has been speculated that an adjuvanted vaccine formulation of IPV would reduce cost and also increase the number of available IPV doses worldwide. In support of this, it was recently shown that the potency of Sabin inactivated polio vaccines is increased when adjuvanted with Aluminium hydroxide or CpG [15], [16]. CAF01 is a novel adjuvant composed of cationic liposomes DDA (dimethyldioctadecylammonium) stabilized with the synthetic immunomodulator TDB (trehalose 6,6′-dibehenate) [17]. CAF01 has proven to enhance both humoral and cell-mediated memory immune responses to a number of different experimental vaccine candidates [17]–[19] in preclinical models. CAF01 has furthermore already been tested in three phase-I trials with an excellent safety and immunogenicity profile (EAG, personal communications and [20]–[22]). Additionally, CAF01 was also found to provide dose-sparing when used in a combination with the trivalent influenza split vaccine in ferrets [23]. We therefore tested the suitably of the CAF01 adjuvant for inducing protective immunity against an infection with polio virus.

It is well known that mucosal immunity can be achieved through vaccination at the mucosal site in question and that a vaccine given by the intramuscular route primarily produce systemic immunity [24], [25]. Mucosal sites are not isolated immunological sites, and priming of an immune response at one mucosal site may also induce protection at another mucosal site [26]. Although the skin is not traditionally regarded as a mucosal site, previous results from other groups have nevertheless indicated that an intradermal administration may confer intestinal/mucosal protection [27], [28]. In addition, intradermal administration is a well-known delivery route for non-adjuvanted IPV vaccines in humans, and has been shown to be a safe delivery route with no adverse effects [29], [30]. We therefore decided to test different vaccine strategies involving both intradermal and intramuscular administration of CAF01 adjuvanted IPV in order to achieve intestinal immunity with an IPV vaccine.

Taken together, the aim for the present work was twofold: 1) to achieve dose sparing with CAF01, measured by neutralization titers, and 2) to induce intestinal immunity against IPV with an IPV-CAF01 vaccine.

## Materials and Methods

### Ethics statement

Experiments were conducted in accordance with the regulations set forward by the Danish Ministry of Justice and animal protection committees by Danish Animal Experiments Inspectorate Permit 2004-561-868 (of January 7, 2004) and in compliance with European Community Directive 86/609 and the U.S. Association for Laboratory Animal Care recommendations for the care and use of laboratory animals. The experiments were approved by the Statens Serum Institut IACUC. The method of sacrifice was cervical dislocation.

### Animal handling

Studies were performed with 6- to 8-wk-old female CB6F1 C57BL/6xBALB/c mice from Harlan, Scandinavia. Animals were housed in appropriate animal facilities at Statens Serum Institut.

### Mono- and trivalent IPV

The IPV vaccines applied throughout this study was commercial GMP grade material manufactured at Statens Serum Institut (SSI). The trivalent IPV was formulated in the ratio 40∶8∶32 for polio virus type 1 (Brunhilde strain, IPV1), type 2 (MEF-1, IPV2) and type 3 (Saukett, IPV3), respectively. The trivalent IPV for the vaccines was in this study always formulated to a trivalent stock, IPV TP (400∶80∶320 DU/mL), from individual concentrated bulk materials of IPV1, IPV2 and IPV3. Monovalent IPVs were stored in M199 buffer.

Dilution of IPV to the respective concentration used for immunization was performed with 10 mM Tris buffer, pH 7.6. The trivalent stock IPV TP of 400∶80∶320 DU/mL was diluted to the appropriate concentration by adding the IPV stock solution to a given volume of buffer. Indicated dosage units in the all experiments correspond to IPV1 D antigen units and all vaccine are trivalent unless otherwise stated.

### IPV-CAF01 formulation

The CAF01 adjuvant suspension was manufactured at two lipid concentrations: 3 mg/mL (CAF01 2500/500 µg of DDA and TDB) and 12 mg/mL (CAF01 10000/2000), respectively. DDA and TDB were manufactured by Niels Clauson-Kaas A/S, Farum, Denmark. In brief, a lipid film for 80 mL CAF01 10000/2000 was formed by dissolving DDA (0.80 g) in 10 mL chloroform/methanol (9∶1, v/v) and mixing it with TDB (0.16 g) dissolved in 10 mL chloroform/methanol (9∶1, v/v) in a 100 mL Bluecap flask. The organic solvent was removed using a gentle stream of N_2_ forming a thin lipid film at the bottom of the flask. The lipid film was dried under vacuum over night to remove trace amounts of the organic solvent. The lipid film was hydrated 80 mL 10 mM Tris-buffer, pH 7.6 by high speed high-shear mixing (Heidolph Silent Crusher M) at 60–80°C for 15 minutes. Adjuvants were stored at 2–8°C until use. For the formulation of CAF01 2500/500, the same procedure is applied, with smaller amounts of DDA and TDB, 0.20 g and 0.04, respectively in 80 mL. All adjuvants were stored at 2–8C until use.

The IPV-CAF01 vaccines were formulated by add-mixing 1∶1 v/v of an IPV solution into a solution of CAF01 under moderate stirring using magnetic stirring bars. The vaccines were rested for 15 minutes to allow for adsorption of IPV to the cationic CAF01 liposomes.

For IPV-CAF01 vaccines intended for mice, the CAF01 10000/2000 µg was applied in order to formulate a mouse dose CAF01 (250/50 µg) into 50 µL dose volume for IM vaccination after 1∶1 v/v mixing with IPV antigen solution. CAF01 2500/500 was applied to formulate vaccines for physic-chemical analyses. All vaccine formulations were performed in LAF units. All vaccines were stored at 2–8C until use. Manufacturing of both the CAF01 adjuvants, IPV vaccines and IPV-CAF01 vaccines was performed in LAF units.

### Immunization

The final concentration of CAF01 in all the mouse studies was 250/50 ug DDA/TDB. Mice were immunized two or three times at two-week intervals intramuscularly (IM in the caudal thigh) with 50 μL IPV or IPV formulated with CAF01. Alternatively the mice were immunized with 50 μL via the intradermal (ID, on the back) route. The IPV doses used in the experiments are indicated in the figures. Two weeks after the second or third vaccination the immune response against IPV was analyzed. In some experiments the immune response was also analyzed 8 and 11 weeks after the second vaccination.

### Lymphocyte cultures

Lymphocytes were cultured and purified as described previously [31]. Briefly, PBMCs were purified on a density gradient and splenocyte cultures were obtained by passage through a cell strainer (BD Pharmingen). IPV for stimulation were all used at 2 μg/mL. Supernatants from triplicate cultures (2×10^5^ cells per well) were harvested from cultures after 72 h of incubation for the investigation of cytokines.

### Cytokine ELISA

A sandwich ELISA was used to determine the concentration of IFN-γ in culture supernatants as previously described [31]. Alternatively, the cytokines were analyzed by Multiplex cytokine assay.

### Multiplex cytokine assay

The Th1/Th2 cytokine 9-plex assay and the IL-17 singleplex assay (Meso Scale Discovery, Gaithersburg, MD) were performed according to the manufacturer's instructions. The plates were read on the Sector Imager 2400 system, and calculation of cytokine concentrations in unknown samples was determined by 4-parameter logistic non-linear regression analysis of the standard curve.

### IPV D-antigen ELISA (DU ELISA)

IPV D-antigen was quantified using a sandwich ELISA. The DU ELISA detects the D-unit activity of IPV. In brief, Immuno plates (Maxisorp, Nunc) were coated with polyclonal antibodies (raised in rabbits by immunization with inactivated poliovirus) of type 1 (Brunhilde strain), type 2 (MEF strain), or type 3 (Saukett strain)) against polio type 1, 2, or 3 diluted in PBS with phenol-red for 2–3½ hours in a humidified atmosphere at room temperature. After washing and blocking of the wells, standards and samples diluted in PBS containing NaCl, KCl, Triton X-100, BSA and phenol-red were added to the wells followed by an overnight incubation in a humidified atmosphere at 2–8°C. The wells were washed and subsequently HRP-conjugated polyclonal antibodies against polio type 1, 2, or 3 diluted in FBS with phenol-red were added to the wells and incubated for 2 hours in a humidified atmosphere at room temperature. The wells were washed and OPD substrate was added followed by a 30 minute incubation period in the dark at room temperature. The reaction was stopped by the addition of H_2_SO_4_ and plates were read at OD 490 nm with non-specific absorption at 630 nm subtracted.

### Microneutralization assay performed at SSI

Briefly, neutralizing antibody titers to the three poliovirus serotypes 1, 2 and 3 were measured using Brunhilde, MEF-1, and Saukett virus stocks on a Vero cells.

Dilutions of the sera in 50 µL were made in duplicate starting at 1∶8 with serial 2-fold dilutions.

50 µl of approximately 100 CCID_50_ (Cell culture infectious dose 50%) of poliovirus type 1, 2 or 3 was added to each well (in a 96 well plate), and incubated for 4½–6 hours at 37 (36–38) °C and 5% CO_2_, and at 2–8°C till the next day. Vero cells were prepared as a cell suspension of 6×10^4^ cells/ml and 50 µL are added to each well containing the polio virus/serum mixture. Plates were incubated at 37°C and 5% CO_2_ for 7 (6–8) days. The wells showing neutralizing/cytopathogenic activity are recorded. The result from a single dilution series is given as 1/√2 times the lowest dilution factor with dead vero-cells and the final titre calculated as the geometric mean of the results from two independent dilution series.

### Microneutralization assay performed at CDC

Samples were tested in triplicate using a standard microneutralization assay for antibodies to poliovirus types 1, 2, and 3 according to established protocols at the Global polio Specialized Laboratory, Centers for Disease Control and Prevention (CDC). Briefly, 80–100 CCID50 of each poliovirus serotype and two-fold serial dilutions of serum were combined and pre-incubated at 35°C for 3 hours before addition of HEp-2(C) cells. After incubation for 5 days at 35°C and 5% CO2, plates were stained with crystal violet and cell viability measured by optical density in a spectrophotometer. Each specimen was run in triplicate, with parallel specimens from one study subject tested in the same assay run, and the neutralization titers estimated by the Spearman-Kärber method and reported as the reciprocal of the calculated 50% endpoint. Each run contained multiple replicates of a reference antiserum pool starting at a 1∶32 dilution to monitor performance variation. A serum sample was considered positive if antibodies were present at ≥1∶8 dilution. Specimens with antibody titers <1∶8 were considered seronegative and specimens with titers >1∶8 were considered seropositive.

### Measurements of antibody titers

Mice were bled for the collection of serum following the vaccination 1–3. Maxisorp micro titer plates (Nunc, Maxisorp, Roskilde, Denmark) were coated with IPV TP (Trivalent inactivated Polio virus, see materials and methods) or IPV Type 1, 2 or 3 (1 ug/mL) in PBS over night at 4°C. Free binding sites were blocked with 2% skimmed milk in PBS. Individual mouse sera were analyzed in duplicate in five-fold dilutions in PBS containing bovine serum albumin starting with a 100-fold dilution. Horseradish peroxidase (HRP)-conjugated secondary antibodies (rabbit anti-mouse IgG, IgG1, IgG2a, IgG2b, IgG2c and IgA; Zymed) diluted 1/2000 in PBS with 1% bovine serum albumin were added. After 1 h of incubation, antigen-specific antibodies were detected by TMB substrate as described by the manufacturer (Kem-En-Tec, Copenhagen, Denmark). To stop the reaction, 100 uL of 4 N H_2_SO_4_ was added, and the optical density (OD) was measured at 450 nm. The absorbance values were plotted as a function of the reciprocal dilution of serum samples.

### Preparation of fecal pellets for antibody analysis

Fecal pellets were collected from mice two weeks following each immunization. The mice were placed in individual cages and fresh fecal pellets (5–6 pellets per mouse) were collected into microfuge tubes containing 600 µL of ice-cold buffer: PBS with soybean trypsin inhibitor (Sigma; 0.1 mg/mL), bovine serum albumin (BSA; 1% w/v), ethylenediaminetetraacetic acid (EDTA; 25 mM), glycerol (50% v/v) and phenylmethylsulfonylfluoride (PMSF; 1 mM). The fecal pellets were broken up to form a suspension and then incubated on ice for 4 hours. After incubation, fecal pellet solutions were clarified by centrifugation at 15,500 G for 10 minutes at 4°C, and the supernatants transferred to microfuge tubes that had been blocked overnight with PBS containing 1% (w/v) BSA. Supernatants were then frozen and analysed at a later date by ELISA.

### Zeta potential and particle size distribution by DLS

The z-average diameter, Z_avg_, of the liposomes was determined by dynamic light scattering using the photon correlation spectroscopy (PCS) technique. The measurements were performed at 25°C using a Malvern nanoZS (Malvern Instruments, Worcestershire, UK) equipped with a 633 nm laser and 173° detection optics. Malvern DTS v. 5.10 software was used for data acquisition and analysis. NanosphereTM size standards 60 nm (Duke scientific corp., Duke, NC) was used to verify the performance of the instrument. The samples were diluted with 10 mM Tris-buffer at pH 7.4 to achieve the optimal vesicle concentration. Surface charge on the vesicles was measured indirectly via analysis of zeta-potential at 25°C using a Malvern nanoZS (using monomodal analysis model) (Malvern Instruments, Worcestershire, UK) of a 1/10-1/400 dilution in milli-Q water.

### Statistical methods

A significant difference in immune responses measured by ELISA, or a difference in neutralization titers, was evaluated using a one-way ANOVA, and Tukey's Multiple Comparison Test for multiple comparisons. A value of *p<*0.05 was considered significant. Prism version 5 software (GraphPad) was used for analysis.

## Results

### Formulating IPV and IPV-CAF01

**Figure 1:**
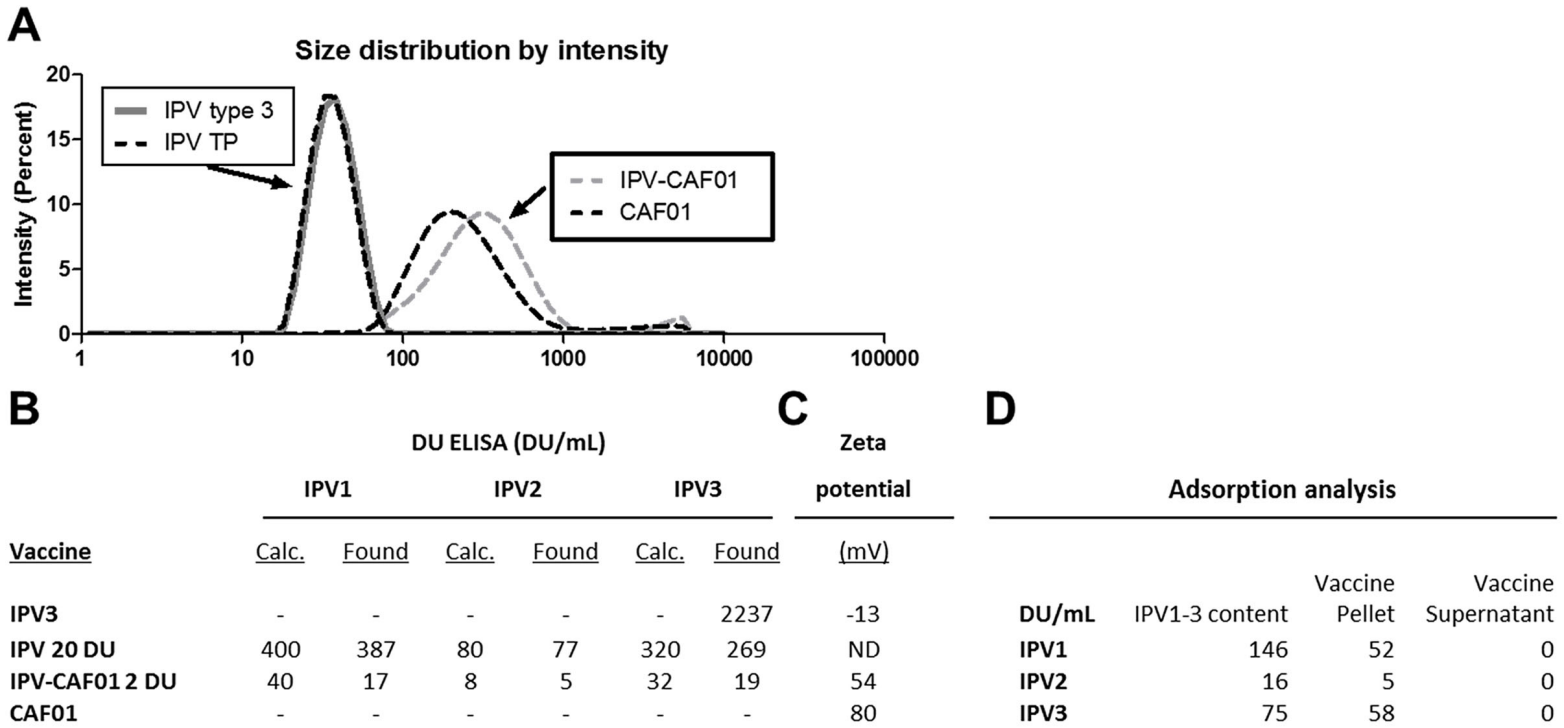
The IPV-CAF01 formulation. **A**. The Z_avg_ hydrodynamic diameter of IPV3 (2237 DU/ml), IPV TP (400∶80∶320 DU), CAF01 (2500DDA/500TDB) and IPV (8DU)-CAF01 (2500DDA/500TDB). All data show the average of 3 measurements. **B**. Analysis of IPV content, as D-unit activity, of the indicated formulations. **C**. The Zeta potential of IPV, CAF01 and IPV-CAF01 formulations. **D**. The degree of association between CAF01 and IPV was determined by measuring the IPV content in the pellet or supernatant fraction by DU ELISA. The content of IPV in similar vaccine without CAF01 was also measured (“IPV1-3 content”).

The trivalent IPV was analyzed with or without CAF01. The trivalent IPV vaccine was formulated from individual IPV1, IPV2 and IPV3 materials. Both IPV3 and the stock IPV TP showed a Z_avg_ (hydrodynamic diameter) of 35 nm. The Z_avg_ of 35 nm is in accordance with values for polio virus size determined by electron microscopy (30 nm) indicating that the size did not change upon mixing of three IPV monovalents into a trivalent IPV (Fig. 1A). Furthermore, the analysis of IPV content measured as D-unit activity confirmed that IPV D-unit activity was maintained after mixing of IPV1-3 into IPV TP (Fig. 1B). On formulation with CAF01, the particle size distribution for an IPV-CAF01 vaccine (Z_avg, d.nm_) was found to be 267 nm compared to 203 nm for the CAF01 adjuvant. This indicated a slight increase in particle size on IPV binding to the CAF01, which however was within an acceptable range.

We also measured the Zeta potential of IPV, CAF01 and IPV-CAF01 formulations (Fig. 1C). The Zeta potential for the cationic CAF01 of 80 mV was in accordance with previous observations [32]. Monovalent IPV showed a slightly negative zeta potential (−13 mV), whereas the adjuvanted IPV-CAF01 vaccine showed a positive zeta potential of 54 mV. Therefore, in IPV vaccines formulated with CAF01, a binding between antigen and adjuvant is electrostatically favorable.

To measure the degree of association between CAF01 and IPV we assumed that since CAF01 is a suspension of cationic liposomes, it is possible to separate an IPV-CAF01 vaccine formulation into a vaccine pellet fraction and a supernatant fraction by centrifugation, and subsequently measure IPV (by DU ELISA) in both fractions. IPV bound to CAF01 will be expected to be located in the vaccine pellet. Three mono-IPV-CAF01 vaccines were formulated to evaluate the binding of each IPV type 1–3. The results of the DU ELISA from centrifuged samples are shown in Fig. 1D. No IPV was found in the supernatant fraction of the three mono-IPV-CAF01 formulations, which indicated that all IPV serotypes are indeed bound to CAF01. This was in agreement with the Zeta potential analysis that suggested a favorable interaction of CAF01 and IPV. On analyzing the resuspended vaccine pellet a recovery rate of 30–75% IPV was obtained (Fig. 1D). The reason for a recovery of less than 100% is most probably due to Steric hindrance (caused by CAF01) of the binding of the detecting antibodies in the IPV DU ELISA to IPV. However, we cannot fully exclude that some IPV was partly inactivated in the CAF01 formulation.

### CAF01 induced dose sparing

We next examined the IPV-CAF01 formulation in mice. The first objective was to determine the ability of CAF01 to increase the neutralizing antibody titer against polio virus in the blood of vaccinated animals. Based on neutralization titers obtained from mice vaccinated with a range of doses from 30 DU (D-Units) to 0.1 DU (data not shown) we choose 20 DU as a full mouse dose and 2 DU as the dose formulated into the CAF01 adjuvant (indicated dose units in the all experiments correspond to polio virus type-1 D antigen units). Thus, the aim was to achieve 10 times dose sparing with CAF01. Mice were immunized with 20 DU IPV or with the lower 2 DU IPV, the latter with or without CAF01. The mice received two immunizations spaced by two weeks and neutralizing antibody titers were determined in the sera two weeks after the last immunization.

**Figure 2:**
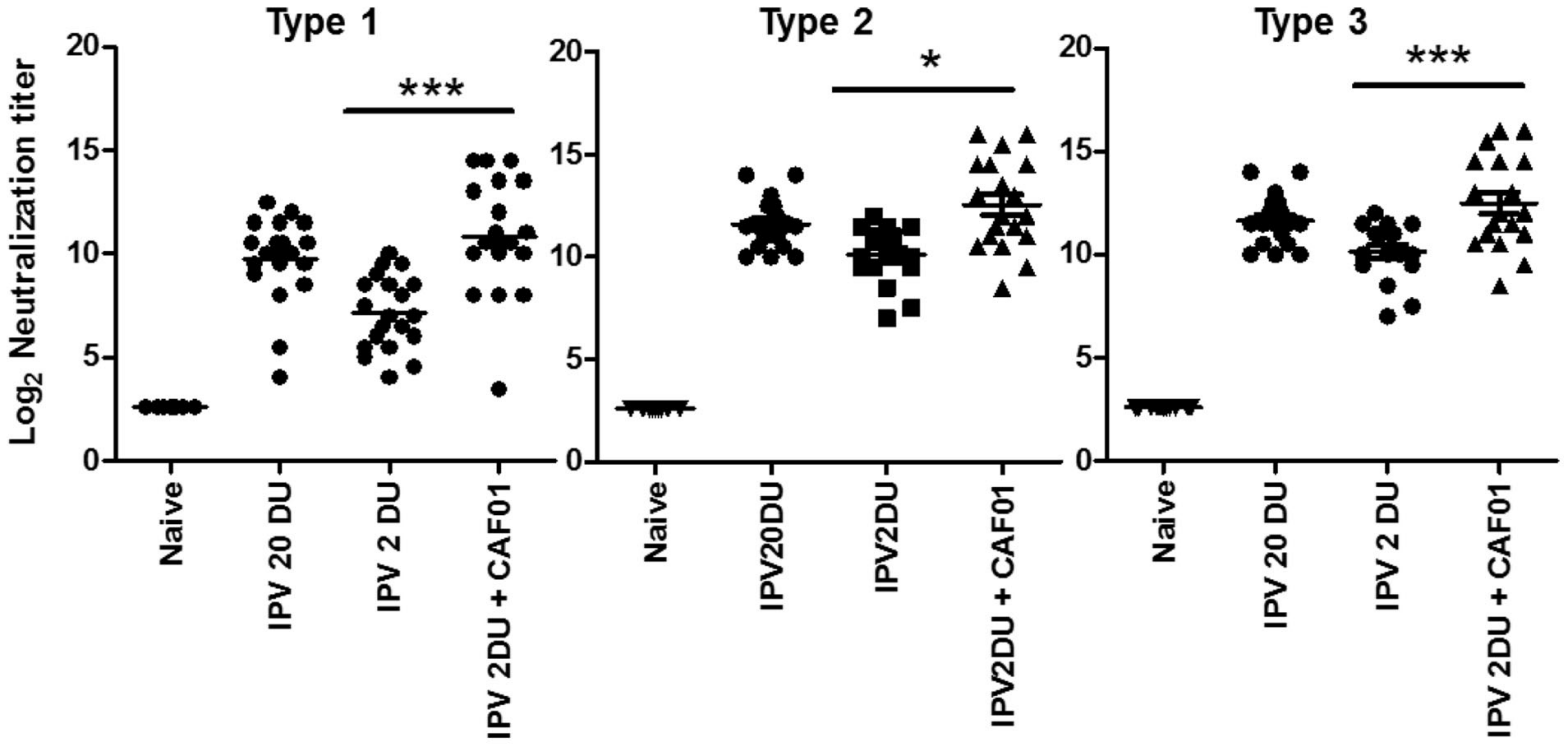
CAF01 induced dose sparing measured by virus neutralization titers. Virus neutralization antibody titers from mice (n = 11–12/group) 14 days following the second IM immunization with inactivated polio vaccine (TP) (with or without adjuvant). Neutralizing antibody titers against poliovirus serotypes 1–3 are shown. The individual titers for each mouse are plotted and the bar represents the mean neutralizing antibody titer. SEM of estimated log_2_ values are shown, *, p<0.05, **, p<0.01, ***, p<0.001 as indicated in the graph using one-way ANOVA and Tukeys post-test for multiple comparisons. Indicated dosage units in the experiments correspond to Type 1 D-antigen units.

Three independent experiments were performed and two assays (performed at Statens Serum Institut, SSI, and Center for Disease Control, Atlants, CDC) were used to determine the neutralization titers. Figure 2 represents all three experiments, whereas the individual experiments are shown in Figure S1. The results showed that CAF01 induced a significant increase in neutralization titers to all serotypes (Type 1, Type 2 and Type 3, p<0.05) (Fig. 2). Neutralization titers to type 1 increased from 7.1+/−1.8 (Log2 transformed titers) in the 2 DU IPV group to 10.8+/−2.7 in the IPV 2DU + CAF01 group. Neutralization titers in the IPV 20 DU group were 9.7+/−2.0 demonstrating a 10× dose sparing effect of CAF01 regarding Type 1 titers. The same patters was seen with Type 2 (IPV 2DU:11.30+/−1.5, IPV 2DU + CAF01: 13.25+/−2.01, IPV 20 DU: 12.12+/−1.82) and Type 3 ((IPV 2DU:10.03+/−1.13, IPV 2DU + CAF01: 12.80+/−2.10, IPV 20 DU: 11.50+/−1.29).

**Figure 3:**
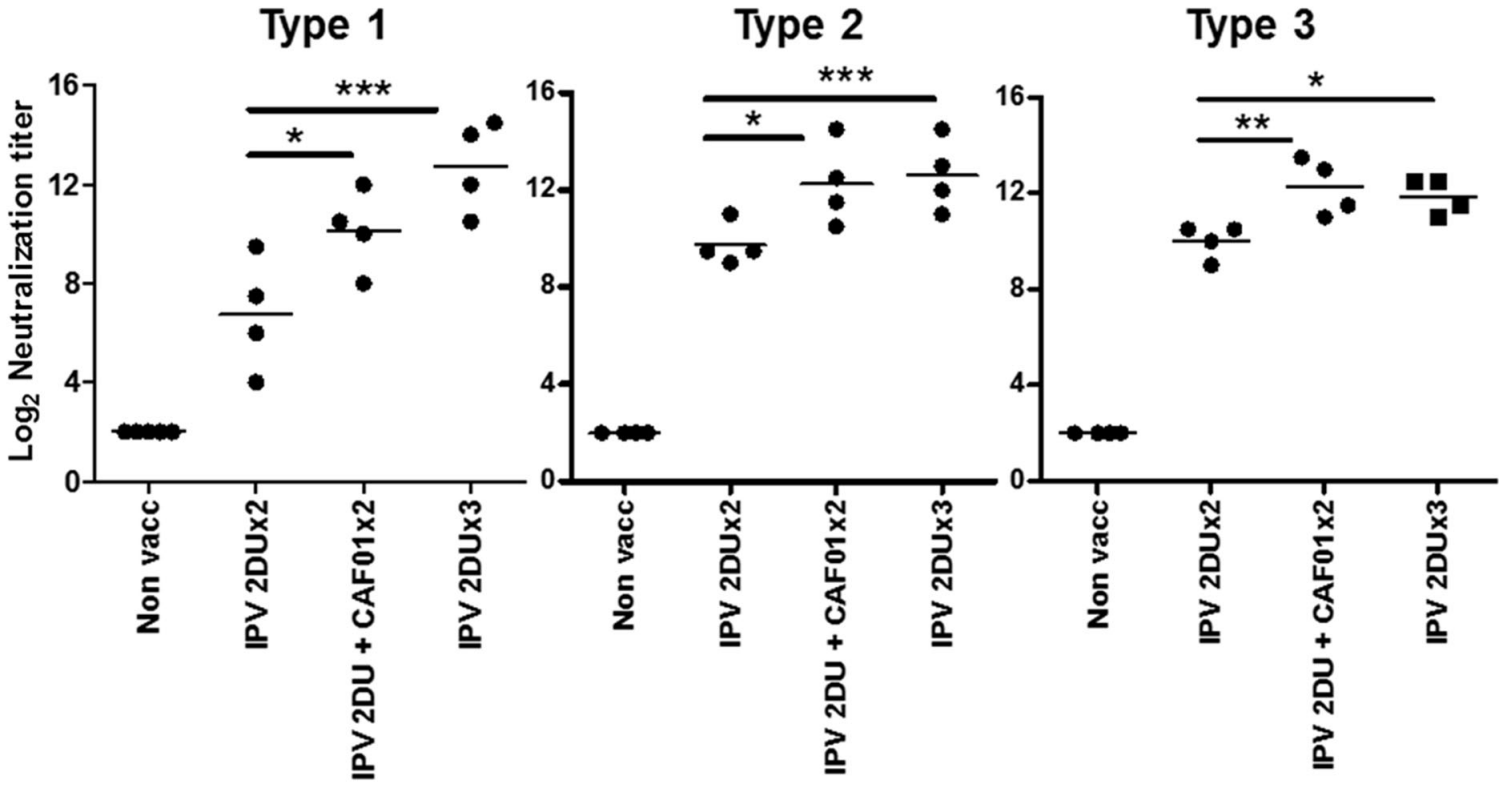
Comparing 2 versus 3 vaccinations. Virus neutralization antibody titers from mice (n = 4/group) 14 days following the second or the third IM immunization with inactivated polio vaccine (TP) (with or without adjuvant). Neutralizing antibody titers against poliovirus serotype 1–3 are shown. The individual titers for each mouse are plotted and the bar represents the mean neutralizing antibody titer. SEM of estimated log_10_ values from N = 4 mice per group *, p<0.05, **, p<0.01, ***, p<0.001 are indicated in the graph using one-way ANOVA and Tukey's post-test for multiple comparisons. Indicated dosage units in the experiments correspond to Type 1 D-antigen units.

A third vaccination led to a significant increase in neutralization titers with the IPV 2 DU group showing an increase from 6.16+/−2.03 to 11.81+/−1.85 (Type 1), 10.75+/−1.34 to 11.94+/−1.24 (Type 2), 10.25+/−1.44 (Type 3) (Fig. 3). However, two vaccinations with IPV 2 DU + CAF01 gave neutralization titers that were not statistically different from 3 vaccinations with IPV 2DU, although there was a trend towards higher titers against type 1 in the IPV alone group that received three vaccinations (Fig. 3).

In conclusion, addition of the adjuvant CAF01 allowed for a 10 times dose sparing. Moreover, only two vaccinations with the CAF01 formulated vaccine were required to achieve the same neutralization titers as observed after three vaccinations with non-adjuvanted IPV.

### CAF01 induced increase in IgG binding titers

**Figure 4:**
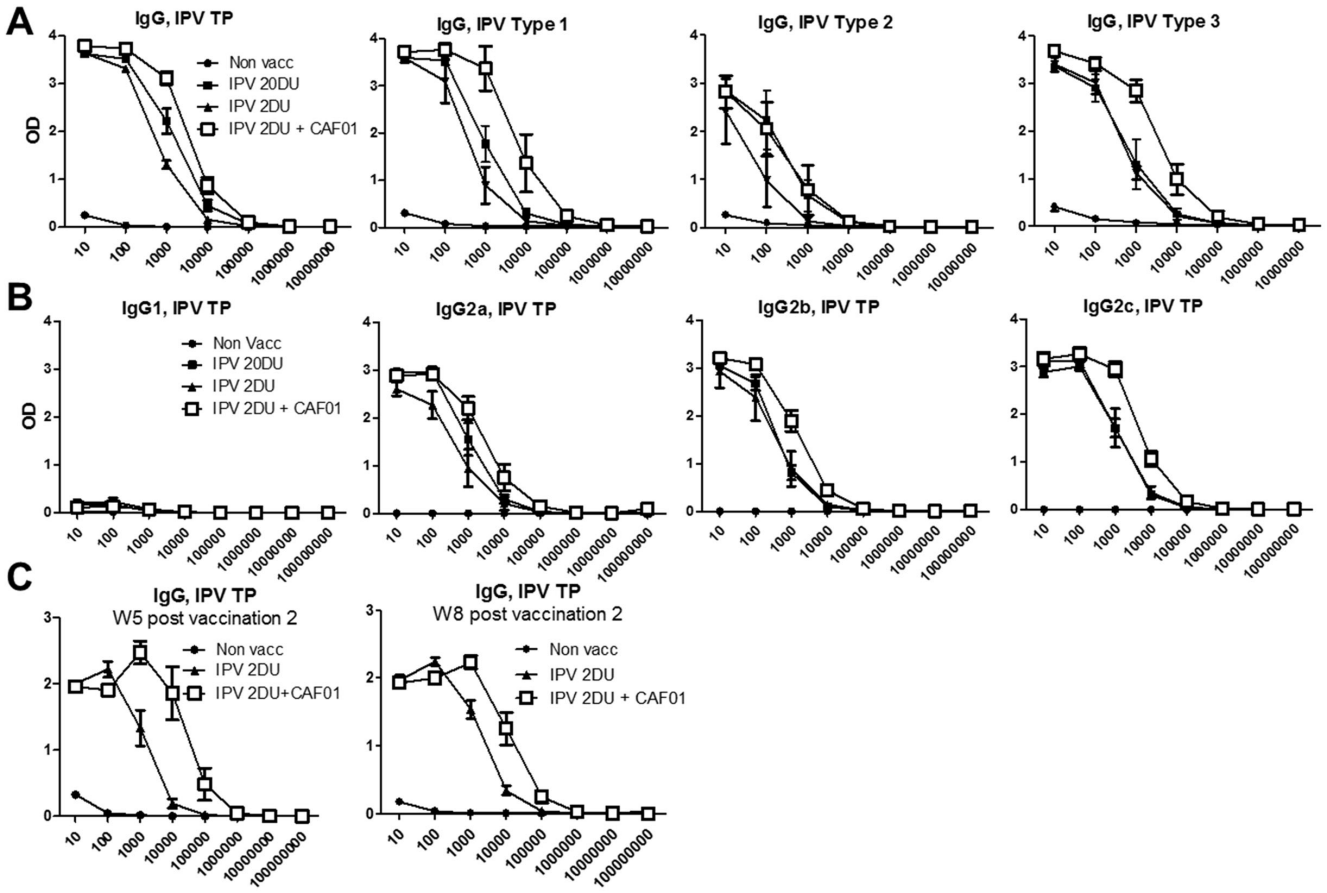
CAF01 increase the Antibody binding titers. **A**. The serum IgG against TP or the individual virus serotypes was measured by indirect ELISA using trivalent IPV or the MPs as the antigen. The Ab titer was measured by the reaction of a series of 10-fold dilution of sera with the antigen. The Ab titration from the sera of 4 mice are shown for the vaccine groups indicated. **B**. The serum IgG1, IgG2a, IgG2b and IgG2c against TP was measured by indirect ELISA using trivalent IPV as the antigen. (N = 6–8). **C**. Serum IgG against TP 5 and 8 weeks post the second vaccination in the vaccination groups indicated (N = 6). Indicated dosage units in the experiments correspond to Type 1 D-antigen units. All mice were vaccinated via the IM route with inactivated polio vaccine (TP).

We also examined if the observed increase in neutralization titers correlated with an increase in the antibody binding titer. Two weeks after the second vaccination, sera from individual mice were analyzed for IPV specific antibody titers measuring both the different IPV specific IgG isotypes (IgG1, IgG2a, IgG2b, IgG2c), as well as the total IgG level against the three virus serotypes. In the IPV 20 DU group we observed higher IgG titers than in sera from the IPV 2 DU against trivalent IPV, or against serotypes 1 and 2. Binding titers against Type 3 were not different between the IPV 20 and 2 DU groups. Importantly, the titers in sera from mice vaccinated with IPV 2 DU + CAF01 were higher than both the IPV 2 DU group against all the serotypes, and at least as high as the IPV 20 DU group (Fig. 4A). The IgG isotypes responsible for the binding titer observed in figure 4A was also examined. The results showed that CAF01 led to increased antibody titers for the IgG isotypes IgG2a, IgG2b, and IgG2c (Fig. 4B). The increased IgG titer observed in the CAF01 group was also observed 5 and 8 weeks following the second vaccination, demonstrating that it was not merely a transient increase (Fig. 4C).

### CAF01 increase the cellular response

To further characterize the IPV-CAF01 formulation we also analyzed the cellular response against IPV. Cellular immunity towards polio virus has received less attention due to compelling evidence that humoral immunity is the most important effector mechanism against polio. However, there are numerous examples that cellular immunity, besides being an effector mechanism in itself, can be important for humoral immunity [33]–[39], even humoral immunity against polio virus [40], [41].

**Figure 5:**
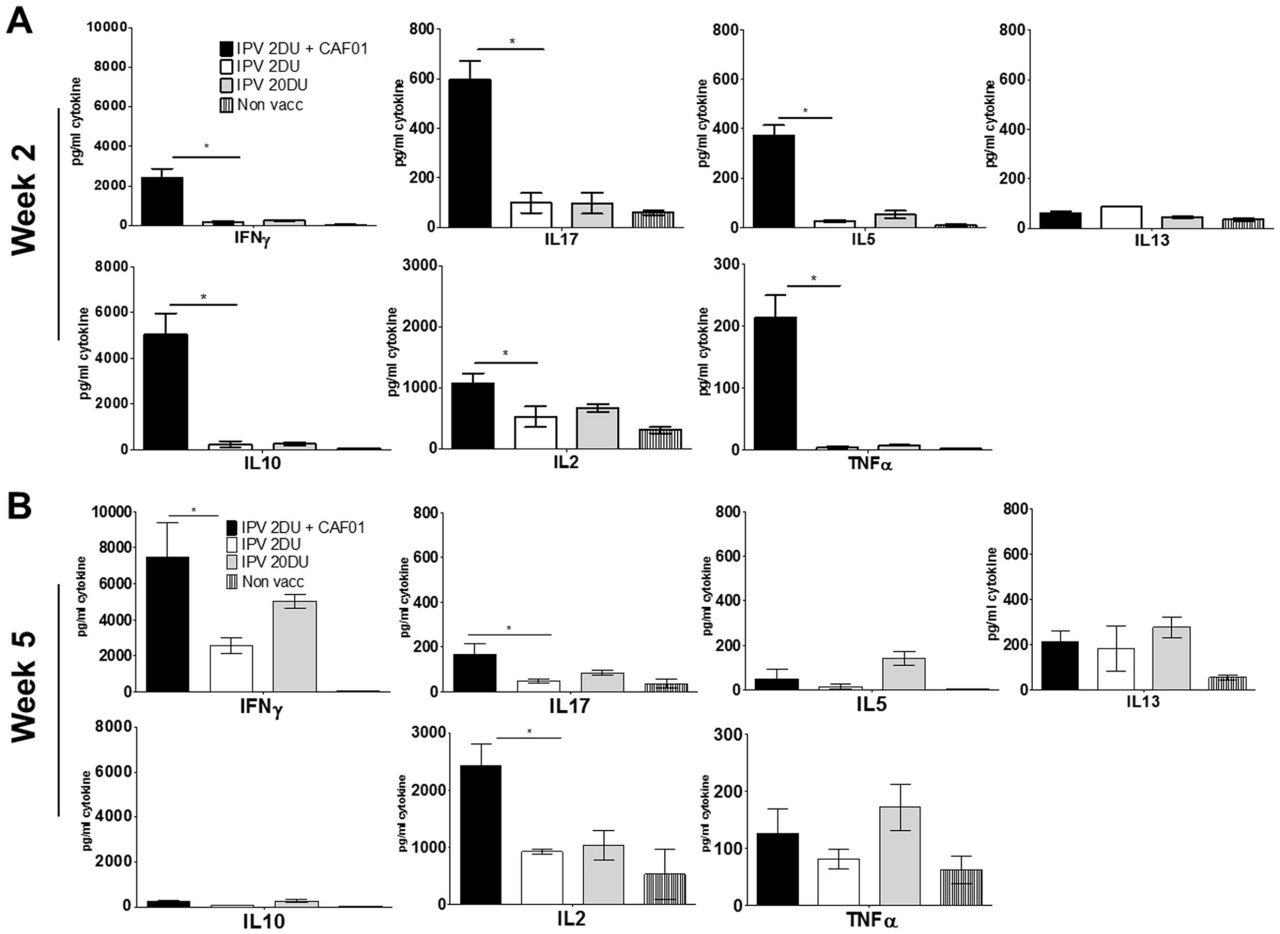
CAF01 induction of cellular immunity. **A and B**. Mice were vaccinated twice at two weeks intervals with IPV (TP) 2DU (white bars), IPV 20DU (grey bars) or IPV 2DU + CAF01 (black bars). Striped bars indicate non-vaccinated mice. At week 2 (A) or week 5 (B) post vaccination 2, PBMCs were stimulated with IPV for 72 hours and secretion of the indicated cytokines was analyzed by MSD. The cytokines are indicated in the figure. Indicated dosage units in the experiments correspond to Type 1 D-antigen units. All mice were vaccinated via the IM route with inactivated polio vaccine (TP).

The cellular response was characterized by analyzing the cytokine profile of IPV-specific T cells induced by vaccination with IPV alone or IPV adjuvanted with CAF01. PBMCs were obtained from mice vaccinated with 2 or 20 DU IPV alone or IPV 2 DU + CAF01 two and five weeks following the booster vaccination. Non-vaccinated mice were included as control. Thereafter, the cells were stimulated in vitro with IPV for 72 hours and the supernatant was collected for Multiplex cytokine analysis. At week 2 post vaccination, in the IPV non-adjuvanted group, Multiplex cytokine analysis showed very low responses for both doses (Fig. 5A). The highest response was observed with IL-2 (525±165 and 667±65 pg/mL in the IPV 2 DU and 20 DU groups, respectively). In contrast, in the IPV 2 DU + CAF01 group we observed a significant increase for the cytokines IFN-γ, IL-17, IL-5, IL-10, IL-2 and TNF-α but not IL13. In particular, at week 2 post vaccination CAF01 led to strong induction of IFN-γ (2379+/−490 pg/mL versus 153+/−86 pg/mL in the IPV 2 DU group) and IL-10 (5024+/−927 pg/mL versus 232+/−138 in the IPV 2DU group).

At week 5 post vaccination, in the IPV alone groups we observed an increased secretion of cytokines IFN-γ (to 2536+/−443 pg/mL in IPV 2 DU mice and 5018+/−394 pg/mL in the IPV 20DU group) and IL-2 (928+/−35 pg/mL in the IPV 2DU mice and 1046+/−255 pg/mL mice). In the IPV 2 DU + CAF01 we observed an increase in IFN-γ (to 7492+/−1896 pg/mL) and IL-2 (to 2432+/−383 pg/mL), but a significant (p<0.05) decrease in secretion of IL-17, IL-5, IL-10, and TNF-α compared to week 2 post vaccination. Thus, at week 5 the major secretion of cytokines was observed with IFN-γ and IL-2 in all the groups with the strongest responses observed in the CAF01-adjuvanted group (Fig. 5).

Taken together, the use of CAF01 induced a faster and stronger cellular response compared to non-adjuvanted IPV. Interestingly, the cytokine profile of the CAF01 group changed from week 2 to week 5 to resemble a Th1 profile similar to the profile induced by IPV alone (Fig. 5).

### A CAF01 vaccine strategy to induce intestinal IgA

Shedding of polio virus is considered the principal marker for protection and studies have implicated intestinal IgA as one of the effector mechanisms to reduce shedding of virus [42]. We therefore examined different vaccine strategies with CAF01 with the aim of generating intestinal IgA. More specifically, as previous experiments have indicated that an intradermal vaccine administration may induce intestinal IgA [27], [28] we decided to first compare intramuscular (IM) and intradermal (ID) administration.

**Figure 6:**
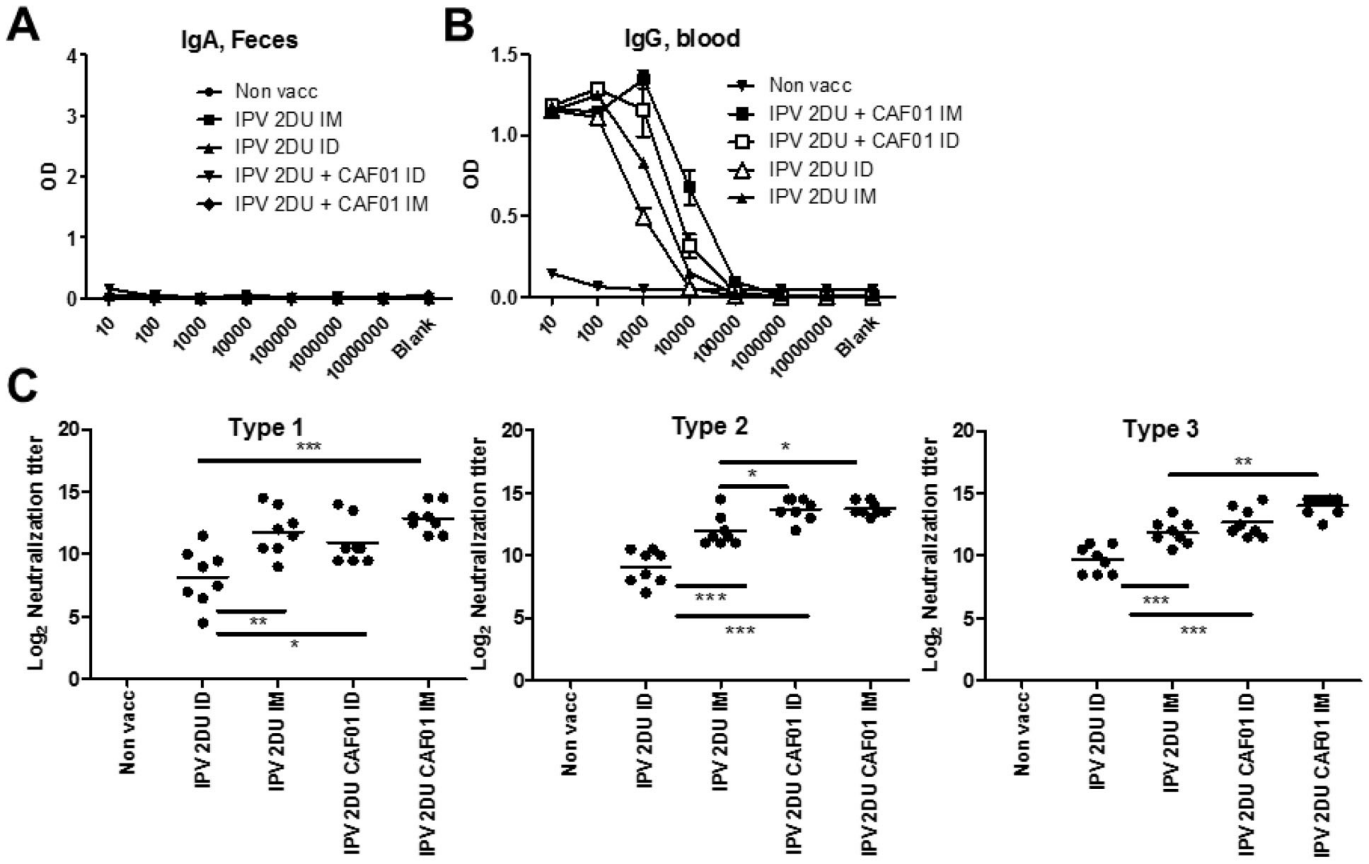
ID versus IM administration. **A**. Mice were vaccinated twice at two weeks intervals. Two weeks after the second immunization with the indicated vaccines IgA was analyzed in fecal samples. **B**. Serum IgG against TP two weeks post vaccination 2 n the vaccination groups indicated (N = 4). **C**. Virus neutralization antibody titers from mice (N = 8) 14 days following the second immunization with inactivated polio vaccine (with or without adjuvant). Neutralizing antibody titers against poliovirus serotype 1–3 are shown. The individual titers for each mouse are plotted and the bar represents the mean neutralizing antibody titer. SEM of estimated log_10_ values from N = 8 mice per group *, p<0.05, **, p<0.01, ***, p<0.001 are indicated in the graph using one-way ANOVA and Tukey's post-test for multiple comparisons. Indicated dosage units in the experiments correspond to Type 1 D-antigen units.

We first examined the IgA production in the intestine two weeks following two vaccinations with either IPV 2 DU + CAF01 given either ID or IM. To avoid the risk of blood contamination of the intestinal samples we only examined fecal pellets taken from the colon of sacrificed animals. Neither of the administration forms induced intestinal IgA (Fig. 6A). In contrast, both vaccine strategies induced IgG in the serum with the IM administration being the highest IgG inducer (Fig. 6B). This correlated well with the serum neutralization titers (Fig. 6C). Thus, IPV-CAF01 induced a significant increase in titers compared to both the IPV ID group and the IPV IM group. This was observed with all the virus serotypes. Furthermore, the ID administration gave a reduced binding and neutralization titer compared to IM administration, suggesting that the ID administration is less efficient at priming a protective humoral immune response compared to the IM administration.

**Figure 7:**
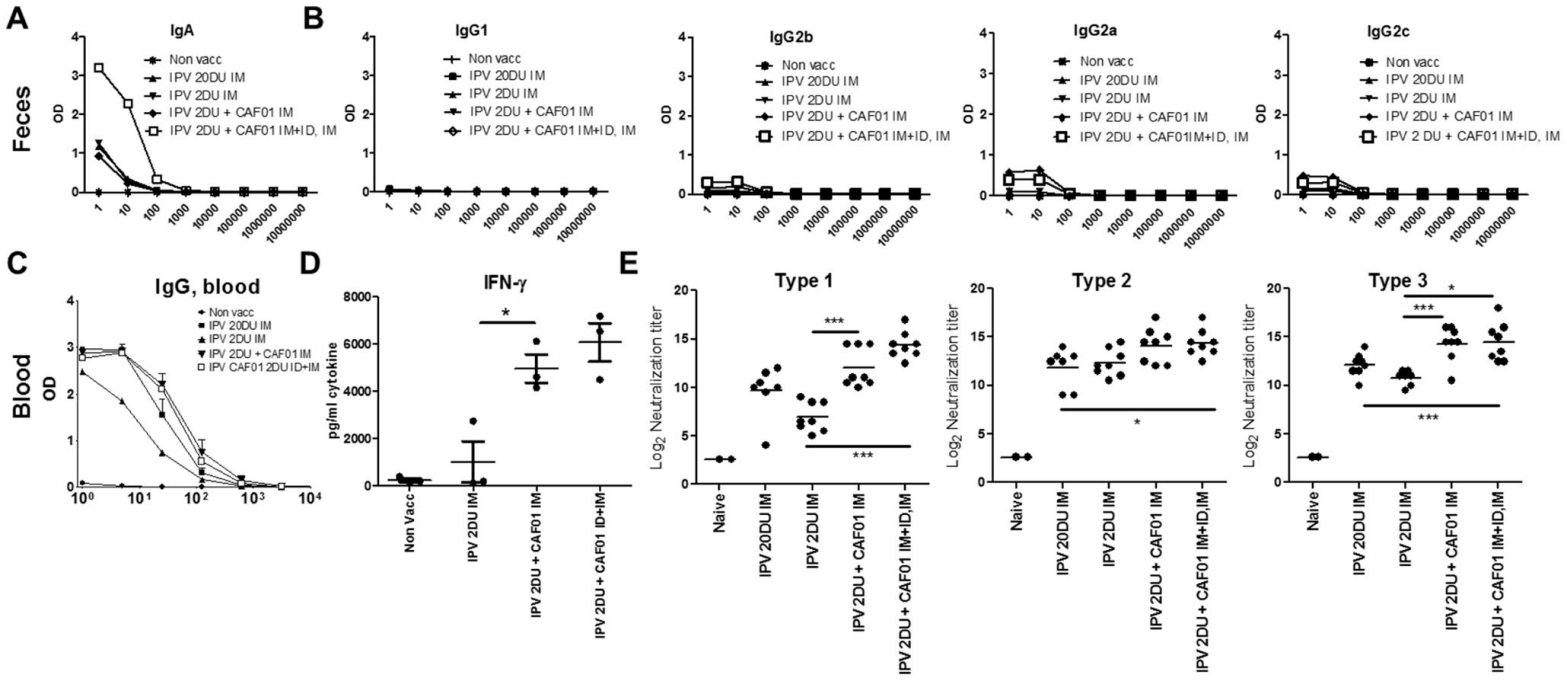
IM+ID administration of CAF01 induces intestinal IgA. **A**. Mice were vaccinated twice at two weeks intervals. Two weeks after the second immunization with the indicated TP vaccines IgA and IgG isotypes was analyzed in fecal samples. The fecal samples were pooled within each experimental group. **B**. Serum IgG against TP two weeks post vaccination 2. The vaccination groups are indicated (N = 4). **C**. Virus neutralizing antibody titers against poliovirus serotype 1–3 are shown. The individual titers for each mouse are plotted and the bar represents the mean neutralizing antibody titer. SEM of estimated log_10_ values from N = 8 mice per group *, p<0.05, **, p<0.01, ***, p<0.001 are indicated in the graph using one-way ANOVA and Tukey's post-test for multiple comparisons. Indicated dosage units in the experiments correspond to Type 1 D-antigen units.

We next decided to test a strategy combining IM administration and ID administration. Of practical importance the animals received 50 µL of the same vaccine in the IM and ID dose (in contrast to two individually formulated vaccines). As booster vaccination the mice were given an IM administration (or another ID+IM co-administration, data not shown). The results showed that supplementing an ID administration with an IM administration in the first vaccination induced a strong increase in the IgA titer in the feces (Fig. 7A). In contrast, we did not observe increased fecal IgG levels (Fig. 7B).

We next examined if the ID+IM administration also affected the systemic humoral or cellular response. At week 2 post the final vaccination the animals were sacrificed and blood was analyzed for IgG binding titers, neutralization titers, and for cellular immunity. IgG titers in both the adjuvanted groups were increased compared to the non-adjuvanted IPV 2DU group and reached levels comparable to the IPV 20 DU group (Fig. 7C). Cellular immunity was measured by IFN-γ ELISA on supernatants from PBMCs stimulated with IPV *in vitro*. The results showed that the IFN-γ response in the IPV 2 DU + CAF01 ID+IM group was as high (6071+/−1404 pg/mL) as in the IPV 2 DU + CAF01 IM group (4960+/−1029 pg/mL) and significantly higher than in the non-adjuvanted group (Fig. 7D). Finally, the neutralization titers in the IPV 2 DU + CAF01 ID+IM group was as high as in sera from the IPV 2 DU + CAF01 IM group, and significantly higher that the non-adjuvanted IPV 2 DU group (Fig. 7E). This was observed for all serotypes, (Type 1: 6.93+/−1.52 versus 12.06+/−2.04, Type 2: 12.31+/−1.41 versus 14.38+/−1.38, Type 3: 10.78+/−0.75 versus 14.50+/−1.96). In fact, titers from the IPV 2 DU + CAF01 ID+IM group were even significantly higher than titers from the IPV 20 DU group demonstrating a dose sparing effect of more than 10 for the IPV 2 DU + CAF01 ID+IM group.

Taken together, compared to IM administration a side by side ID+IM administration with a CAF01- adjuvanted vaccine followed by an IM administration did not negatively affect systemic immunity, measured by antibody binding and neutralization titers and T cell IFN-γ secretion (Fig. 6). Importantly, in contrast to the IPV 2 DU ID or IPV 2 DU IM groups, in the IPV 2 DU ID+IM group we observed a significant increase in fecal IgA whereas fecal IgG was not observed in either of the vaccine groups. As the ID+IM group received twice the antigen amount in the first vaccination, we also tested a group that received two IM vaccinations at the same time (at two different sites). However, this did not lead to fecal IgA (data not shown). Finally, we did not observe any significant adverse effects at the ID site of administration.

## Discussion

The primarily goal of the present study was to examine if the adjuvant CAF01 could be used to achieve dose sparing with IPV, and a subsequent goal was to achieve intestinal immunity with an IPV-CAF01 vaccine.

### Achieving dose sparing with CAF01

It is well known that a prerequisite for worldwide replacement of OPV with IPV is the availability of affordable IPV in low and middle-income countries. Lower cost options being pursued include reducing the number of doses and the amount of antigen per dose [15], [16], [43], [44]. In addition, lowering the manufacturing cost for IPV in developing countries is a priority. Our results showed that with the adjuvant CAF01 we were able to achieve a 10 times dose sparing effect in mice measured by the virus neutralization titers in the blood (Fig. 2). Importantly, the results also showed that two vaccinations with IPV 2 DU + CAF01 gave neutralization titers that either equaled those induced by 3 vaccinations with IPV 2 DU (Type 2 and 3 titers), or were slightly reduced (Type 1 titers) (Fig. 3). Thus, addition of the adjuvant CAF01 induced a significant dose sparing effect, or allowed for a vaccine schedule with one less dose. The potential of applying only two vaccinations compared to three will have significant economical and practical impact.

Presently, IPV vaccination schedules involve up to 5 doses commonly administered at week 6, 10, 14 and at month 9 and 15. A recent report concluded that children in resource-poor settings probably are protected against paralytic poliomyelitis with as few as two doses of IPV in primary vaccines [45]. The study was based on IPV vaccines given at month 6 and 9, i.e. when maternal transferred antibodies have declined and consequently were not expected to inhibit the IPV vaccines. In addition, children receiving a previous OPV vaccination were protected with just a single IPV dose most probably due to boosting of a prominent OPV induced memory B cell response [45]. Thus, at least with a booster vaccine, the study indicated that one or two vaccinations should protect children in resource-poor settings against paralytic poliomyelitis [46]. Our results indicated that the inclusion of an adjuvant will affect both the required dose (allowing for a 10× reduced IPV dose) and the number of vaccinations (allowing for a vaccine schedule with less vaccine doses) required to induce proper protection against infection with polio virus.

The response to IPV and several other vaccines are highly sensitive to the maternal antibody level [47]–[52]. There were significantly lower rates of seroconversion for all three poliovirus types in a study performed in Thailand and for types 1 and 3 in an Oman study including babies from birth and 24 weeks onwards and with children with baseline maternal antibody titres of ≥64. In addition, previous studies have shown that IPV appears to be more sensitive than OPV to the effect of high maternal antibody titers on reducing the neutralizing antibody response to vaccine given in the first months of life [28]–[32]. However, previous studies have highlighted two important points concerning the antibody response against IPV. One study showed that maternal antibodies inhibited vaccine-induced antibody responses but not T cell responses, and that the latter allowed for high antibody titers following a booster vaccine [53]. In addition, Krishnan et al. [54] showed that the negative effect of maternal antibodies on the response to IPV may be reduced by increasing the intervals between doses. Taken together, and in combination with our results, we suggest that with the inclusion of an adjuvant, such as CAF01, combined with a booster vaccine given at a longer interval than the commonly used 4 weeks (e.g. 8 weeks as proposed previously [54]), a 5–10-fold reduction of the IPV vaccine dose is indeed feasible, even in the presence of maternal antibodies.

### The CAF01 induced immune response against IPV

We also characterized the influence of CAF01 on the immune response against IPV. Firstly, the antibody binding titers at week 2 post vaccination 2 were increased to levels that in most cases were higher than the levels induced by a 10 times higher IPV dose (20 DU) (Fig. 4). This was observed against all three polio virus types (Fig. 4A). Interestingly, in contrast to for IgG2a, IgG2b, and IgG2c, we only observed a minor IgG1 response in both adjuvanted and non-adjuvanted groups (Fig. 4B). In contrast, previous observations with the CAF01 adjuvant formulated with recombinant proteins showed a strong IgG1 response [55]. This suggested that even in the presence of a strong adjuvant, IPV can influence the IgG response. As the IgG isotype profile of the IPV-CAF01 vaccine resembled that of IPV alone, this suggested that it is IPV that determines the IgG profile even in the presence of CAF01.

The increased antibody response induced by CAF01 also correlated with an increased cellular response measured by multiplex cytokine analysis. Although the cellular immune response is not considered a direct correlate of protection for polio vaccines it may play an indirect role by supporting a memory B cell response [56], [57]. Our analysis of anti-IPV T cell immunity revealed that CAF01 induced both a faster and stronger response against IPV. At week 2 post vaccination only a minor response was observed in the non-adjuvanted IPV groups in contrast to the CAF01-adjuvanted groups that showed increased levels against all the cytokines tested (Fig. 5A). The CAF01 adjuvanted group also showed elevated cytokine responses at week 5 post vaccination 2. Interestingly our results also showed that the cytokine profile of the IPV-CAF01 group changed significantly from week 2 to week 5 to resemble the profile induced by IPV alone, a profile dominated by IFN-γ and IL-2 secretion (Fig. 5B). This indicates that IPV as an antigen can also influence the type of cellular immunity induced against it. Furthermore, as IFN-γ is known to induce isotype switch to IgG2a, these results also explains the observed induction of IgG2a, and the lack of IgG1. Thus, both regarding the IgG response and the cellular T cell response the role of CAF01 in the formulation with IPV is to induce a response that is quantitatively different from the response induced by IPV but not qualitatively different. The observed T cell profile presented in our study is in agreement with a previous study showing the same cytokine profile following immunization with Sabin IPV +/− Alum [40]. Interestingly, in that study it was also demonstrated that the T cells were required for the protection against a polio infection, most probably by acting as helper T cells for B cells, and through the stimulation of neutralizing antibody production of the IgG2a type. In addition, CD4 T cells have also been shown to be required for the generation of optimal antibody responses following infection with coronavirus [33], vaccinia virus [34], [35], yellow fever virus [36] or vesicular stomatitis virus (VSV) [37], supporting that the role of T cells against a viral infection such as polio virus should not be underestimated. We speculate that T cell immunity mediated by the IFN-γ/IL-2 expressing Th1 T cells may be important for the protection against the polio virus due to their ability to 1) induce isotype switching to IgG2a and 2) induce innate immunity to contributes to antiviral immunity [40], [41], [58].

### Inducing intestinal immune responses with an IPV-CAF01 vaccine

It is accepted that the major port of entry for the polio virus is the intestinal tract, and that fecal IgA is important in preventing entry of the pathogen [42], [59]. Therefore, inducing intestinal IgA is a priority of any polio vaccine.

Several publications indicate an immunological connection between the intradermal and intestinal site [27], [28]. However, in humans, the results obtained with ID administration of IPV are not entirely clear. Thus, one large-scale pediatric study conducted in Cuba by the WHO Initiative for Vaccine Research [60] failed to meet non-inferiority criteria, whereas another showed that three immunizations with one-fifth of the dose of IPV given ID resulted in similar seroconversion rates compared to three full doses of IPV given IM in infants [61] although the antibody were significantly lower in infants given the decreased ID dose of IPV [61]. Thus, the usefulness of the ID administration route for IPV is still being debated.

The results presented in this paper indicated that as an injection site to prime an immune response, the intradermal administration is not as efficient as the IM administration in mice. This was based on experiments showing reduced binding/neutralization titers in the ID group compared to the IM group, receiving the same vaccine (Fig. 6). In addition, the ID administration did not induce IgA in the intestine or in fecal samples (Fig. 6 and data not shown). However, compared to IM or ID alone administration, a side-by-side ID+IM administration with a CAF01 adjuvanted vaccine followed by an IM administration induced significant levels of fecal IgA and, of importance, without compromising serum binding and neutralization titers. Although a subject for future investigations, it may be suggested that T cells primed systemically by IPV-CAF01 can be recruited to other sites, e.g. the intestine, and act as helper T cells to potentiate the response at these sites. This may be of some importance as it suggests that a strong intestinal priming may not be entirely required for intestinal protection as long as an intestinal challenge can effectively boost a pre-existing minor intestinal immunity and/or lead to fast recruitment of immunity residing in other (systemic) locations. In fact, in this respect it is of particular interest that some studies have shown that IPV (administered via the IM route) may indeed ‘prime’ substantial protective intestinal immunity [7]. Furthermore, in tropical developing countries, a supplemental dose of IPV administered to children previously exposed to OPV boosts humoral and intestinal immunity more effectively than a supplemental dose of OPV [5]–[7], [45], [62], which is supported by a study performed in the Netherlands [63]. Thus, it seems reasonable to assume that an IM vaccination with IPV will induce immunity with some ability to interact with the intestinal site and that a simultaneous priming at both the IM and ID site with the same vaccine will increase the intestinal response.

### Concluding remarks

In conclusion, we have for the first time characterized the formulation of IPV with the CAF01 adjuvant. The formulation proved to constitute a stable formulation where the majority of IPV was associated with CAF01 and maintained its D-unit activity. IPV-CAF01 induced a strong systemic protective immunity in mice and also influenced the kinetics of the IPV immune response to produce a faster response. Finally, a simultaneous priming at an intradermal and an intramuscular site with the IPV-CAF01 vaccine led to intestinal immunity. CAF01 has successfully completed several phase I studies with other vaccine candidates, and further studies are now ongoing to support the development the IPV-CAF01 towards a human trial.

## Supporting Information

Figure S1 Virus neutralization titers from 3 individual experiments. Virus neutralization antibody titers from mice (n = 8/group) 14 days following the second or the third immunization with inactivated polio vaccine (with or without adjuvant). Neutralizing antibody titers against poliovirus serotype 1–3 are shown. The individual titers for each mouse are plotted and the bar represents the mean neutralizing antibody titer. SEM of estimated log10 values from N = 4 mice per group *, p<0.05, **, p<0.01, ***, p<0.001 are indicated in the graph using one-way ANOVA and Tukey's post-test for multiple comparisons. Indicated dosage units in the experiments correspond to Type 1 D-antigen units.
(TIF)
