# Supplementary figures and images for: Inducing Dose Sparing with Inactivated Polio Virus Formulated in Adjuvant CAF01
Source: PLoS One. 2014 Jun 23;9(6):e100879. doi: 10.1371/journal.pone.0100879 (PMC4067388; doi:10.1371/journal.pone.0100879)

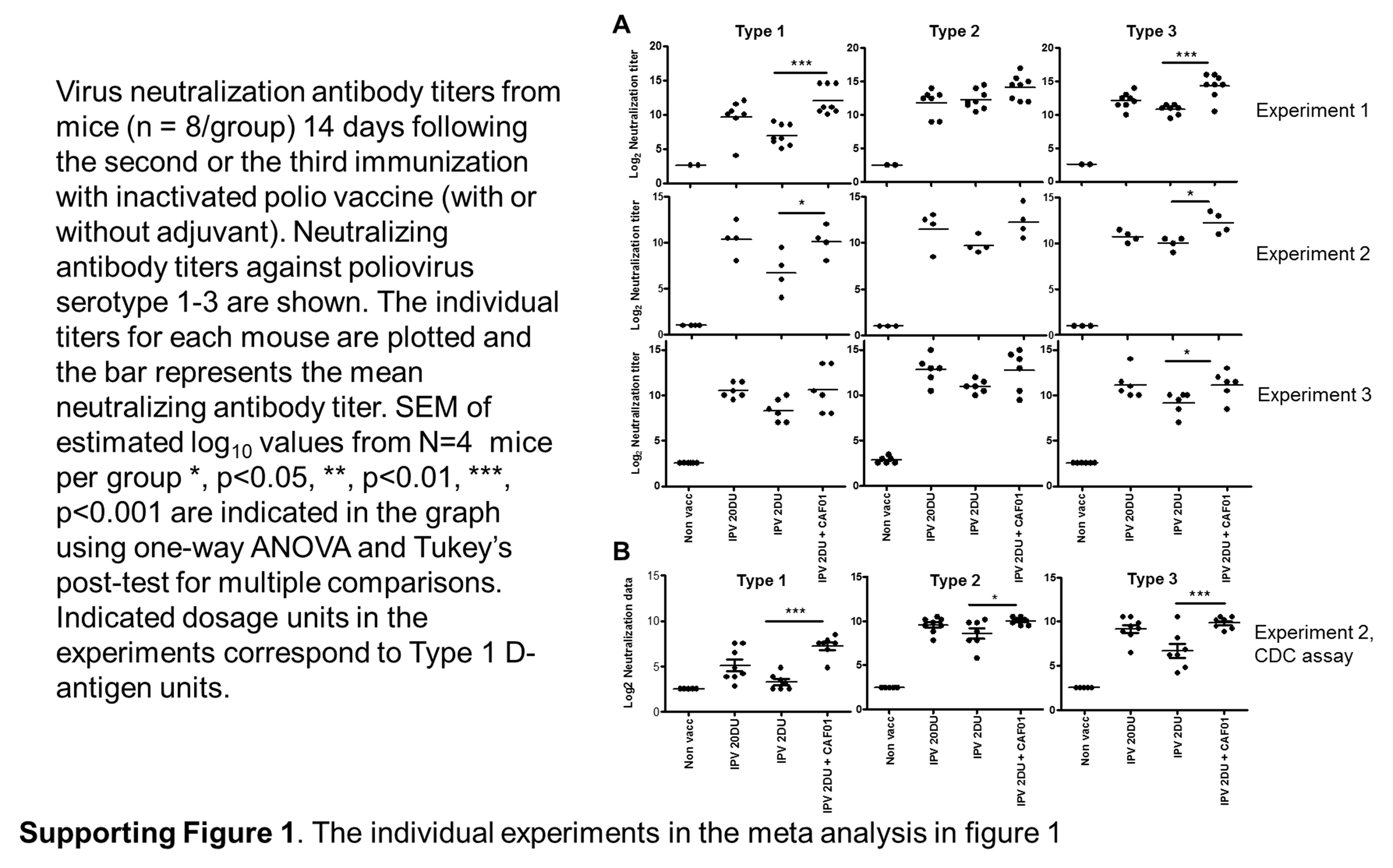

Supplement: Figure S1 — Virus neutralization titers from 3 individual experiments. Virus neutralization antibody titers from mice (n = 8/group) 14 days following the second or the third immunization with inactivated polio vaccine (with or without adjuvant). Neutralizing antibody titers against poliovirus serotype 1–3 are shown. The individual titers for each mouse are plotted and the bar represents the mean neutralizing antibody titer. SEM of estimated log10 values from N = 4 mice per group *, p<0.05, **, p<0.01, ***, p<0.001 are indicated in the graph using one-way ANOVA and Tukey's post-test for multiple comparisons. Indicated dosage units in the experiments correspond to Type 1 D-antigen units. (TIF) [file pone.0100879.s001.tif]
